# Alteration of Extracellular Superoxide Dismutase in Idiopathic Pulmonary Arterial Hypertension

**DOI:** 10.3389/fmed.2020.00509

**Published:** 2020-11-17

**Authors:** Rui Zhang, Lan Wang, Qin-Hua Zhao, Rong Jiang, Su-Gang Gong, Xin Jiang, Xi-Qi Xu, Yang-Yang He, Yuan Li, Zhi-Cheng Jing

**Affiliations:** ^1^Department of Cardio-Pulmonary Circulation, Shanghai Pulmonary Hospital, Tongji University School of Medicine, Shanghai, China; ^2^Department of Cardiology, Peking Union Medical College Hospital, Chinese Academy of Medical Sciences & Peking Union Medical College, Beijing, China

**Keywords:** idiopathic pulmonary arterial hypertension, superoxide dismutases, extracellular SOD, biomarkers, prognosis

## Abstract

**Background:** Superoxide dismutases (SODs) are an important family of antioxidant enzymes that modulate reactive oxygen species levels. It is largely unknown which SOD isoform(s) change *in vivo* in idiopathic pulmonary arterial hypertension (IPAH) patients.

**Methods:** A total of 133 consecutive adult IPAH patients who underwent bone morphogenetic protein receptor type 2 (*BMPR2)* genetic counseling were enrolled in this prospective study. The plasma activities of three subtypes of SOD [copper–zinc (Cu/Zn-SOD), manganese (Mn-SOD), and extracellular SOD (Ec-SOD)] were examined.

**Results:** The activities of SODs were significantly lower in IPAH patients than in healthy subjects. However, only Ec-SOD activity in *BMPR2* mutation patients was significantly decreased compared to those in patients without a mutation. The reduced Ec-SOD activity was markedly associated with mean pulmonary arterial pressure, pulmonary vascular resistance (PVR), and 6-min walking distance (6MWD). The reduction of Mn-SOD activity was only associated with 6MWD. There was no association between Cu/Zn-SOD and hemodynamics. Patients with a lower Ec-SOD level had a worse survival compared to those with a higher baseline. The reduced Ec-SOD activity and the raised PVR increased the mortality risk.

**Conclusions:** Ec-SOD was correlated with *BMPR2* mutation, hemodynamic dysfunction, and poor outcomes. Circulating Ec-SOD could be a potentially vital antioxidant enzyme in the pathogenesis of IPAH.

## Introduction

The enhanced production of superoxide anions and other reactive oxygen species (ROS) contributes to the pathogenesis of pulmonary arterial hypertension (PAH) (1). The superoxide or ROS inactivates endothelium-derived nitric oxide (NO) and promotes the progression of endothelial dysfunction. Accordingly, steady-state levels of superoxide are dependent on both its rate of production and the activity of various antioxidant enzymes (2, 3). Superoxide dismutases (SODs) are one family of important antioxidant enzymes that defend against superoxide radicals in vascular protection. The expression and the activity of SODs presumably have a profound effect on the responses of vascular cells to both acute and chronic oxidative stress (4).

Blood vessels express three isoforms of SODs: copper–zinc SOD (Cu/Zn-SOD, SOD 1), which locates in the cytosol, manganese SOD (Mn-SOD, SOD 2), which locates in the mitochondrial matrix, and an extracellular form of Cu/Zn-SOD (Ec-SOD, SOD 3) (4, 5). In mammals, different SOD isoforms are encoded by distinct genes but catalyze the same reaction. A reduction of SOD activity has been implicated in patients with PAH and in animal models of PAH (1, 6–12). For example, Cu/Zn-SOD knockout mice spontaneously displayed signs of elevated right ventricular systolic pressure and pulmonary arterial remodeling under normoxia (6). Mn-SOD deficiency was likewise evident in pulmonary arteries and plexiform lesions. Tissue-specific, epigenetic Mn-SOD deficiency initiated and sustained a heritable form of PAH by impairing redox signaling and resulting in proliferation and apoptosis resistance of pulmonary arterial smooth muscle cells (PASMC) (7). In a recent phase I and open-label clinical study in IPAH patients, the plasma Mn-SOD level change was used as a potential marker of drug effect (8). Remarkably, both Ec-SOD mRNA expression and activity were decreased in the lung tissue of idiopathic PAH (IPAH) (9). The loss of function or selective depletion of Ec-SOD exacerbated PAH (10, 11). The intratracheal delivery of adenoviruses overexpressing Ec-SOD could suppress monocrotaline-induced PAH in rats (12). Taken together, these data suggested that SODs are important antioxidant enzymes in the pathogenesis of PAH.

Identifying the link between defective SOD activity and clinical characteristics in IPAH patients is important, which, in turn, can be used as an epigenetic biomarker for a new and improved therapeutic strategy. Approximately 20% of patients with IPAH carried mutations in bone morphogenetic protein receptor type 2 (*BMPR2*), where a loss of *BMPR2* function may compromise the integrity of the endothelial barrier and contribute to endothelial dysfunction by mediating endothelium-derived nitric oxide bioactivity (2, 13, 14). To date, there is limited information on plasma SOD alteration in IPAH patients with or without *BMPR2* mutation. Thus, the objective of the present study was to prospectively determine whether (a) the abnormalities of SOD levels were related to hemodynamic dysfunction and clinical characteristics, (b) patients with *BMPR2* mutation had a more severe reduction of SOD levels, and (c) plasma SOD level could be a predictor for prognosis and clinical outcome.

## Materials and Methods

### Study Subjects

One hundred thirty-three consecutive adult IPAH patients (≥18 years of age at diagnosis) who underwent *BMPR2* genetic counseling at the time of their first right heart catheterization were prospectively enrolled in this study between January 2010 and July 2013. One hundred thirty control subjects were selected from a cohort of healthy volunteers. The median age of the control subjects was 40 (20–57) years, and the ratio of women to men was 3:1. IPAH was diagnosed according to standard criteria: mean pulmonary artery pressure (mPAP) ≥25 mmHg and pulmonary vascular resistance (PVR) at rest >3 Wood units, in the presence of a normal pulmonary artery wedge pressure (PAWP ≤15 mmHg) (15). Patients were excluded if they have definite causes for PAH, such as connective tissue disease and congenital heart disease, and also those with portopulmonary hypertension, chronic pulmonary thromboembolism, pulmonary hypertension due to left heart diseases, and lung diseases and/or hypoxemia. Other exclusion criteria for the study included potential confounding factors associated with plasma antioxidant enzyme production: cigarette smoking and excessive alcohol consumption, hypertension, and type 2 diabetes mellitus (16, 17). We also excluded the participants who completed an acute exercise and were with vitamin C and E supplementation (18). We prospectively followed up these patients for a mean of 26 ± 9 months after enrollment, and no patient received lung or heart–lung transplantation. The major endpoint was defined as all-cause mortality.

The study was conducted according to the principles of the Declaration of Helsinki and was approved by the Shanghai Pulmonary Hospital Ethics Committee (number K16-055). Written informed consent was obtained from all the participants.

### Blood Sampling and Plasma SOD Assay

Venous blood was collected from all subjects after fasting overnight (>12 h) to minimize the influence of foods and beverages on the plasma SOD concentrations. All the samples were collected directly into specially prepared sodium ethylene diamine tetra-acetic acid tubes containing a preservative to retard auto-oxidation. After centrifuging at 3,000 rpm at 4 for 15 min, the supernatant was separated for SOD activity immediate determinations. The whole procedure was completed within 20 min. For the oxidant test, the presence of 0.005% butylated hydroxytoluene (without glutathione) in plasma was allowed for anti-*ex vivo* oxidation and improving the stability, routinely. Each sample was measured within a month of collection, and at least two different dilutions of the same sample were tested. To minimize the inter- and the intra-assay coefficients of variation, each analyte was duplicated on three different days within 1 month. The plasma Cu/Zn-SOD and Mn-SOD activities were determined by an SOD assay kit (Cayman Chemical Company, item no. 706002) using a tetrazolium salt reaction (9). The method utilized tetrazolium salt for the detection of superoxide radicals generated by xanthine oxidase and hypoxanthine. Detection of only Mn-SOD activity needs the addition of potassium cyanide to inhibit both Cu/Zn-SOD and Ec-SOD. The samples can be assayed in the absence of xanthine oxidase to generate a sample background. This sample background absorbance (at 440–460 nm) was subtracted from the sample absorbance generated in the presence of xanthine oxidase, thus correcting for non-SOD-generated absorbance.

The plasma activity of Ec-SOD was performed based on the competitive ELISA assay kit (Lifespan Biosciences, NBP1-90377) according to the manufacturer's instructions (19). Each well of the supplied microtiter plate has been pre-coated with a target-specific capture antibody. Standards or samples are added to the wells as well as a fixed quantity of biotin-conjugated target antigen. The antigens in the standards or samples compete with the biotin-conjugated antigen to bind to the capture antibody. An avidin–horseradish peroxidase (HRP) conjugate is then added, which binds to the biotin. A 3,3′,5,5′-tetramethylbenzidine substrate was used for testing for Ec-SOD, and the optical density of the well is measured at a wavelength of 450 ± 2 nm.

### Statistical Analysis

Results were expressed as numbers, percentages, means with corresponding standard deviations, or medians with corresponding 25th and 75th percentiles [interquartile range (IQR)]. Continuous variables were compared with baseline characteristics, hemodynamic parameters, and SOD levels using Student's *t*-test or Mann–Whitney *U*-test according to normality. The proportions were compared with Pearson chi-square test or Fisher's exact test, as appropriate. Continuous variables were assessed for linearity of their relationship with the outcome variable. Spearman's ρ was investigated for correlations, with a Bonferroni correction for each variable. If these variables were not found to be linearly related to the outcome, they were grouped into quartiles and modeled to avoid violating model assumptions. A univariate analysis of covariance adjusted for age and sex was carried out to compare the means among the three groups (control subjects, *BMPR2* mutation carrier group, and BMPR2 wild-type group). Bonferroni method was applied to correct the *p*-value for multiple comparisons in *post hoc* tests.

For comparison of the prognostic values of SODs and selected hemodynamic parameters, receiver operating characteristic curves (ROC) were generated and the areas under the curves were calculated. The optimal thresholds for baseline Ec-SOD for death prediction was determined using Youden index (sensitivity + specificity−1). The value of Ec-SOD corresponding to the maximum value of Youden's index was considered as the optimal cutoff point for Ec-SOD. Survival analyses were performed using Kaplan–Meier method and were compared by means of log-rank test. Two steps were performed to analyze the survival. First, a univariable Cox proportional regression was used for time-to-event analysis to estimate hazard ratios (HR) and 95% confidence intervals (CI) for all-cause mortality according to the stratified covariates (SODs, hemodynamic parameters, 6MWD, WHO FC, female gender, and age). All variables with a *p* < 0.05 were then tested in a stepwise forward Cox regression analyses; the variables were entered at a *p* < 0.05. In the second step of the survival analysis, a multivariable stepwise forward Cox regression model was used to estimate the HR and the 95% CI for association in different SOD groups and outcomes adjusted for age and sex. For all analyses, *p* < 0.05 were considered as statistically significant. All calculations were performed using the SPSS 14.0 statistical software package (Statistical Package for Social Science, Chicago, IL, USA) or StatView 5.0.1 (SAS Institute, Cary, NC, USA).

## Results

### SOD Levels and Characteristics of the Study Population

**Figure 1:**
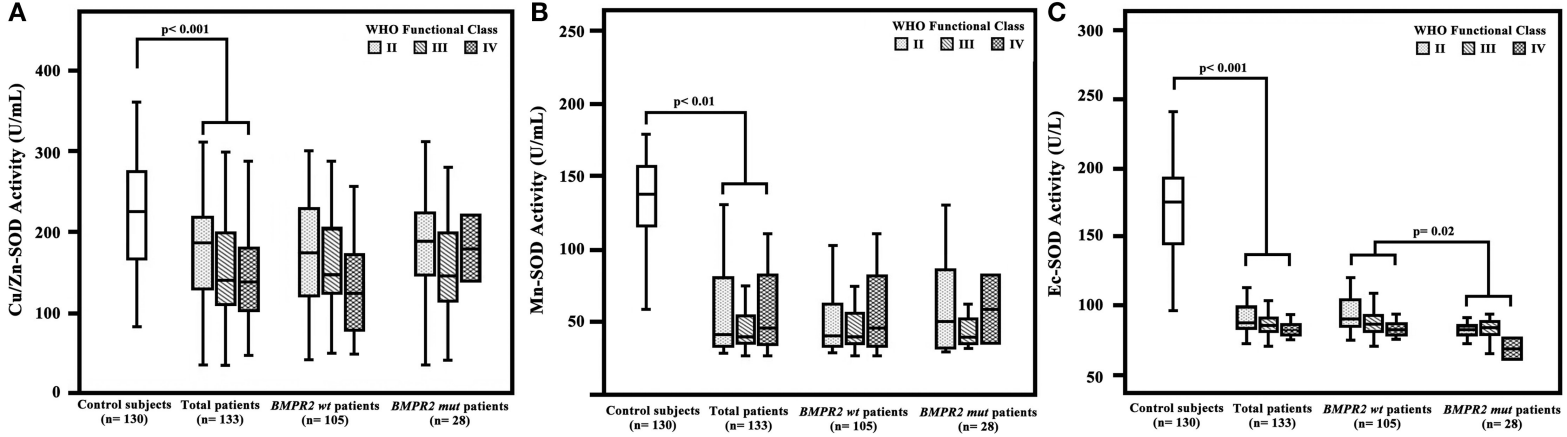
Plasma superoxide dismutase (SOD) activity in patients with idiopathic pulmonary arterial hypertension (IPAH), control subjects, patients with *BMPR2* mutation (*BMPR2* mut), and those with wild-type *BMPR2* (*BMPR2* wt), associated with WHO functional class. **(A)** The plasma Cu/Zn level was significantly decreased in patients with IPAH compared to that in the control subjects. **(B)** The Mn-SOD activity was significantly lower in patients with IPAH compared to that in the control subjects. **(C)** The Ec-SOD activities in patients with IPAH were significantly lower compared to those in the control subjects. The Ec-SOD activity in patients of the *BMPR2* mut group was statistically decreased compared to that in patients of the *BMPR2* wt group. The line through the center of the boxes represents the median. *BMPR 2*, bone morphogenetic protein receptor type 2; Cu/Zn-SOD, copper–zinc superoxide dismutase; Mn-SOD, manganese superoxide dismutase; Ec-SOD, extracellular form of Cu/Zn superoxide dismutase.

Among the 133 IPAH patients recruited, 28 (21%) were *BMPR2* mutation carriers (*BMPR2* mut); the other 105 patients were *BMPR2* wild-type (*BMPR2* wt). The clinical characteristics are summarized in Table 1. The patients with *BMPR2* mut had a younger median age at diagnosis (28 years; IQR, 22–34 years) than those of with *BMPR2* wt (39 years; IQR, 29–53 years; *p* < 0.001). The sex ratio of females to males was 2.4:1 (*n* = 94/39) in the total population. In *BMPR2* wt patients, the female/male ratio was 3.0:1 (*n* = 79/26), whereas in the *BMPR2* mut group, the female/male ratio was 1.2:1 (*n* = 15/13, *p* < 0.001). In patients with IPAH, plasma Cu/Zn-SOD (148 U/ml; 95% CI, 114–213), Mn-SOD (40 U/ml; 95% CI, 34–115), and Ec-SOD (85 U/L, 95% CI, 79–119) activities were significantly lower compared to those in the 130 control subjects (212 U/ml, 95% CI: 153–266; 144 U/ml, 95% CI: 114–157; and 175 U/L, 95% CI: 143–193, respectively; *p* < 0.01 or *p* < 0.001). Among the three comparison groups (control subjects, *BMPR2* mut group, and *BMPR2* wt group), plasma Ec-SOD activity was still lowest in the *BMPR2* mut group, adjusted for female gender (*F* = 6.679, *p* = 0.010) and age (*F* = 1.420, *p* = 0.002). There was no difference of Cu/Zn-SOD and Mn-SOD in the multiple-comparison tests. Only Ec-SOD activity in patients with *BMPR2* mut group was statistically decreased compared to those in the *BMPR2* wt group (*BMPR2* wt, *p* = 0.02, Figure 1). However, there was no significant difference regarding WHO functional class severity in both *BMPR2* mut and *BMPR2* wt groups as well as total patients.

**Table 1:** Baseline characteristics and SOD levels in patients with IPAH.

	**Total (*n* = 133)**	***BMPR2* mut (*n* = 28)**	***BMPR2* wt (*n* = 105)**	***P-*value^a^**
Age, years	36 (27, 51)	28 (22, 34)	39 (29, 53)	<0.001
Female gender, *n* (%)	94 (71)	15 (54)	79 (75)	<0.001
BMI, kg/m^2^	22 (20, 24)	21 (20, 23)	22 (20, 24)	0.163
WHO FC, *n* (%)				0.191
Class II	45 (34)	9 (32)	36 (34)	
Class III	71 (53)	17 (61)	54 (51)	
Class IV	17 (13)	2 (7)	15 (14)	
Onset to diagnosis, months	24 (7, 48)	18 (5, 43)	24 (8, 48)	0.293
6MWD, meters^b^	367 (311, 439)	407 (330, 248)	360 (308, 440)	0.376
BNP, pg/ml	355 (144, 515)	307 (143, 533)	391 (157, 475)	0.812
Cu/Zn-SOD, U/ml	148 (114, 213)	147 (125, 215)	142 (110, 213)	0.608
Mn-SOD, U/ml	40 (34, 65)	40 (34, 80)	40 (34, 163)	0.863
Ec-SOD, U/l	85 (79, 91)	82 (77, 86)	86 (81, 93)	0.010
**Hemodynamic variables**				
HR, bpm	84 (75, 94)	86 (76, 92)	84 (74, 98)	0.787
mRAP, mmHg	7 (5, 12)	5 (7, 12)	7 (5, 12)	0.862
mPAP, mmHg	62 (51, 69)	68 (57, 82)	59 (50, 66)	0.003
PAWP, mmHg	9 (7, 11)	9 (7, 11)	9 (7, 11)	0.959
CO, L/min	3.6 (3.0, 4.7)	3.4 (2.8, 4.7)	3.6 (3.0, 4.7)	0.561
CI, L/min/m^2^	2.2 (1.8, 32.8)	2.0 (1.7, 2,7)	1.9 (1.6, 2.5)	0.362
PVR, Wood units	15 (10, 19)	16 (13, 23)	14 (9, 19)	0.039
S_V_O_2_, %	59 (54, 67)	58 (55, 67)	59 (54, 67)	0.654
**PAH-specific therapies**, ***n*** **(%)^c^**				0.848
Bosentan (oral)	16 (12)	4 (14)	12 (11)	
Iloprost (inhaled)	3 (2)	0 (0)	3 (3)	
Sildenafil (oral)	59 (44)	14 (50)	45 (43)	
Vardenafil (oral)	30 (23)	5 (18)	25 (24)	
Combination therapy	15 (11)	2 (7)	13 (12)	

*Values are expressed as medians (interquartile range), except for female gender and WHO functional class, which are numbers of patients (%)*.

a *Comparison between groups with BMPR2 mut and BMPR2 wt*.

b *6MWD could be successfully measured in 124 patients*.

c *Seven patients (5%) were enrolled in PATENT-1 (Riociguat) study*.

*BMI, body mass index; BMPR 2, bone morphogenetic protein receptor type 2; BNP, brain natriuretic peptide; CI, cardiac index; CO, cardiac output; Cu/Zn-SOD, copper–zinc superoxide dismutase; Ec-SOD, extracellular form of Cu/Zn superoxide dismutase; HR, heart rate; Mn-SOD, manganese superoxide dismutase; mPAP, mean pulmonary arterial pressure; mRAP, mean right atrial pressure; mut, mutation; 6MWD, 6-min walking distance; PAWP, pulmonary artery wedge pressure; PVR, pulmonary vascular resistance; SvO_2_, mixed venous oxygen saturation; WHO FC, World Health Organization functional class; wt, wild-type*.

### Correlation of SOD Activity With Hemodynamic Variables

**Figure 2:**
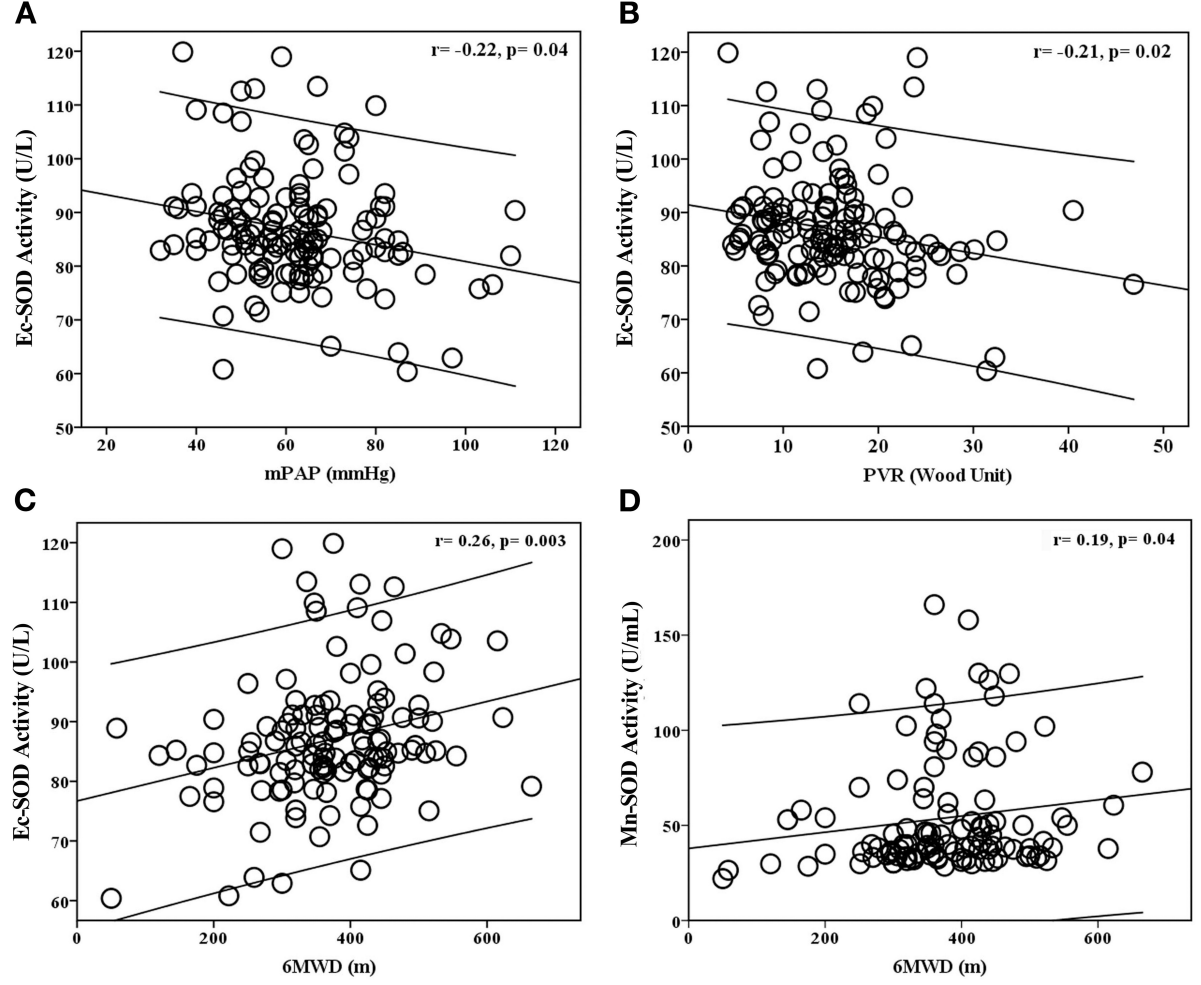
Relationship between baseline plasma Ec-SOD activity with **(A)** mean pulmonary arterial pressure (mPAP), **(B)** pulmonary vascular resistance (PVR), **(C)** 6-min walking distance (6MWD), and **(D)** plasma Mn-SOD activity with 6MWD in patients with idiopathic pulmonary arterial hypertension (IPAH). The baseline plasma Ec-SOD activities were negatively correlated with mPAP and PVR. The Ec-SOD and Mn-SOD activities were correlated positively with 6MWD.

The *BMPR2* mut patients had a more severe hemodynamic compromise, with a significantly higher mPAP and PVR in comparison with the *BMPR2* wt patients (Table 1). The baseline plasma Ec-SOD activities were negatively correlated with mPAP (*r* = −0.22; *p* = 0.04) and PVR (*r* = −0.21; *p* = 0.02). The Ec-SOD and the Mn-SOD activities were correlated positively with 6MWD (*r* = 0.26; *p* = 0.003, *r* = 0.19; *p* = 0.04, respectively, Figure 2). The other baseline SOD activities did not correlate with age, mRAP, and mixed venous oxygen saturation (S_V_O_2_) (Supplementary Figures S1–S8).

### SOD Activities in Relation to Other Markers of Adverse Outcomes

**Figure 3:**
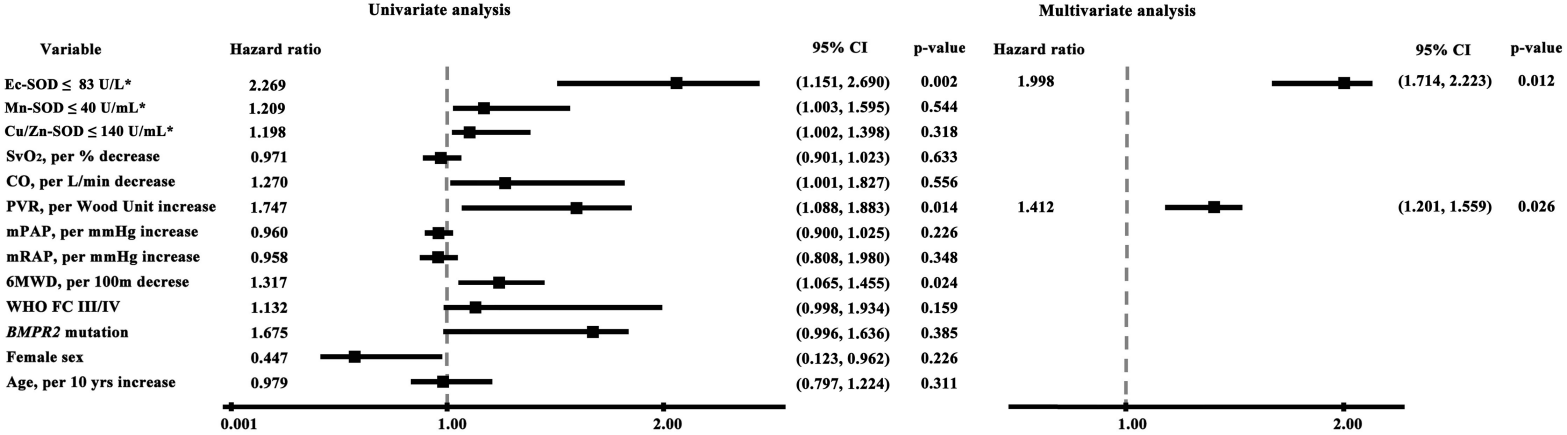
Cox proportional hazards model of factors associated with survival in idiopathic pulmonary arterial hypertension (univariate and multivariate analyses). The lower Ec-SOD and the higher PVR remained as significant predictors of adverse outcomes. The asterisk indicates identification by the receiver operating characteristic analysis curve-derived cutoff value. *BMPR 2*, bone morphogenetic protein receptor type 2; CO, cardiac output; Cu/Zn-SOD, copper–zinc superoxide dismutase; Ec-SOD, extracellular form of Cu/Zn superoxide dismutase; Mn-SOD, manganese superoxide dismutase; mPAP, mean pulmonary arterial pressure; mRAP, mean right atrial pressure; 6MWD, 6-min walking distance; PVR, pulmonary vascular resistance; SvO_2_, mixed venous oxygen saturation.

A univariate analysis identified several factors related to mortality. An elevated mRAP, an increased PVR, and a reduced Ec-SOD were all significantly associated with an increased risk of death. By a stepwise multivariate Cox regression analysis, only the lower Ec-SOD and the higher PVR remained as significant predictors of adverse outcomes (Figure 3).

**Figure 4:**
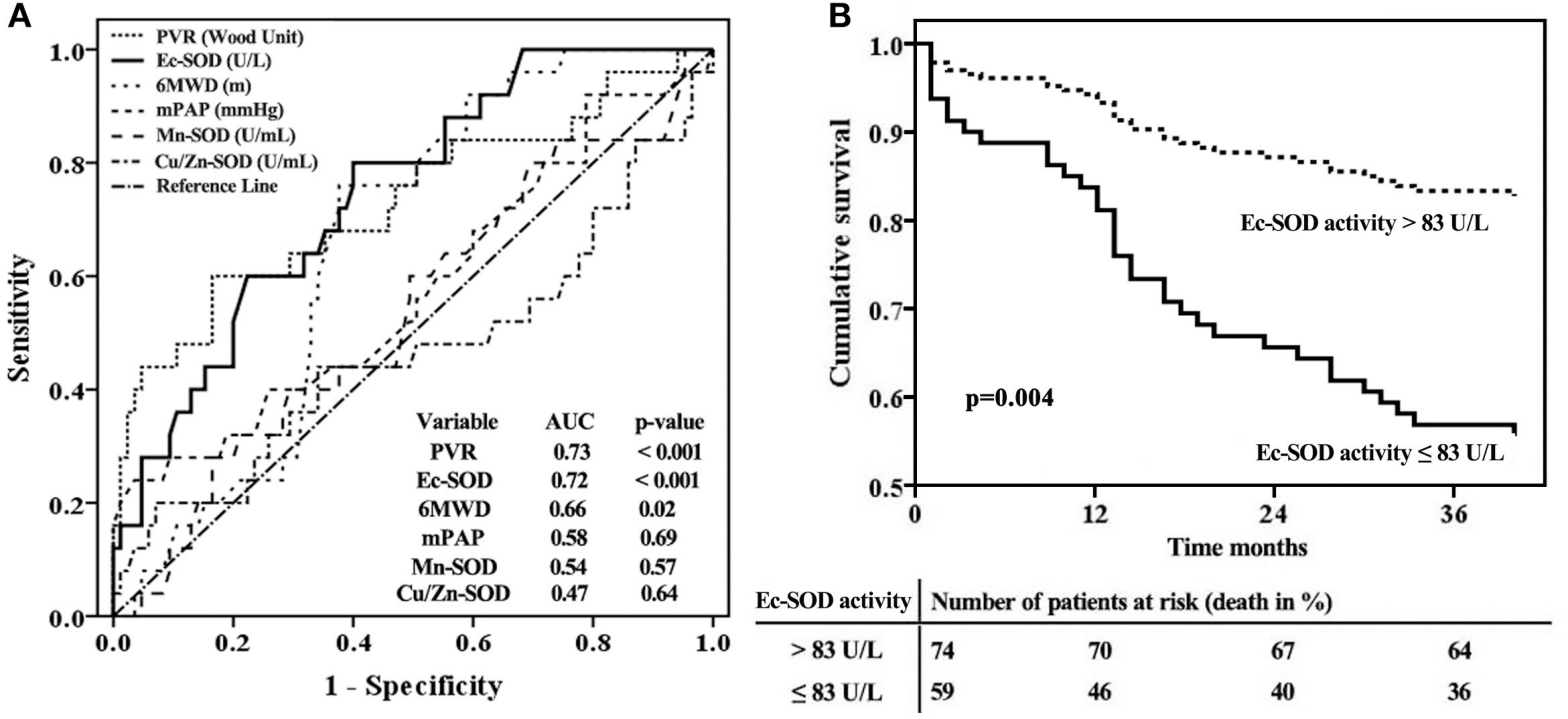
Extracellular form of Cu/Zn superoxide dismutase (Ec-SOD) activity in relation to other markers for an adverse prognosis according to **(A)** receiver operation characteristic analyses and **(B)** survival curves for the baseline cutoff plasma Ec-SOD activity in patients with idiopathic pulmonary arterial hypertension, adjusted by age and female gender. The best Ec-SOD cutoff level for predicting outcome was 83 U/L. Survival was significantly better in patients with Ec-SOD activity >83 U/L.

The ROC curve analyses further illustrated that Ec-SOD was a strong indicator of adverse outcomes in IPAH. The best Ec-SOD cutoff level for predicting outcome was 83 U/L, giving a sensitivity rate of 69% and a specificity rate of 71%. The c-statistic for Ec-SOD level was 0.72 (95% CI, 0.64–0.85), which was similar to PVR (0.73; 0.60–0.86) but numerically superior to 6MWD (0.66; 0.55–0.76), mPAP (0.58; 0.44–0.71), Mn-SOD (0.54; 0.41–0.67), and Cu/Zn-SOD (0.47; 0.32–00.61, Figure 4A).

### Survival Analysis

The observed cardiopulmonary mortality was 26% (35 patients) in the current cohort of IPAH patients. Twenty-five of the deaths were directly related to right ventricular failure; six had a sudden death, and the cause of death was not able to be ascertained in four cases. The total patients with IPAH were divided into two groups based on the cutoff levels for Ec-SOD calculated by ROC analysis to detect mortality.

In the group of 59 patients with Ec-SOD activity ≤83 U/L, 24 (41%) died, but only 11 of 74 (15%) patients were in the group with Ec-SOD activity >83 U/L (*p* < 0.001). The patients with Ec-SOD activity >83 U/L had a significantly lower mPAP and PVR than those with Ec-SOD activity ≤83 U/L. Moreover, the proportion of female gender and the severity of WHO functional class in the group with Ec-SOD ≤83U/L were higher than those with Ec-SOD >83 U/L (Table 2). Survival was significantly better in patients with Ec-SOD activity >83 U/L (Figure 4B). The 1- and 3- year survival estimates were 84 and 63%, respectively, in patients with Ec-SOD activity ≤83 U/L and 95 and 86%, respectively, in patients with Ec-SOD activity >83U/L (*p* = 0.004), adjusted by age and female gender.

**Table 2:** Baseline characteristics in relation to Ec-SOD activity.

	**Ec-SOD ≤ 83 U/L (*n* = 59)**	**Ec-SOD > 83 U/L (*n* = 74)**	***P*-value^a^**
Age, years	31 (25, 48)	39 (29, 52)	0.022
Female gender, ***n*** (%)	45 (76)	49 (66)	<0.001
BMI, kg/m^2^	22 (19, 23)	22 (21, 24)	0.500
BMPR2 mutation	17 (29)	11 (15)	0.521
WHO FC, ***n*** (%)			<0.001
Class II	14 (24)	31 (42)	
Class III	35 (59)	36 (49)	
Class IV	10 (17)	7 (9)	
Onset to diagnosis, months	18 (6, 33)	34 (7, 60)	0.033
6MWD, meters^b^	360 (276, 415)	390 (329, 452)	0.008
BNP, pg/ml	387 (158, 529)	319 (147, 488)	0.857
Cu/Zn-SOD, U/ml	146 (110, 213)	148 (120, 213)	0.911
Mn-SOD, U/ml	46 (34, 90)	39 (29, 52)	0.082
**Hemodynamic variables**			
HR, bpm	82 (73, 94)	86 (76, 97)	0.409
mRAP, mmHg	8 (5, 13)	7 (5, 11)	0.631
mPAP, mmHg	63 (54, 77)	59 (50, 67)	0.048
PAWP, mmHg	9 (6, 11)	9 (7, 12)	0.453
CO, L/min	3.5 (3.0, 4.5)	3.6 (2.9, 5.0)	0.410
CI, L/min/m^2^	2.3 (1.8, 2.8)	2.2 (1.8, 2.8)	0.792
PVR, Wood units	16 (12, 22)	14 (9, 17)	0.040
S_V_O_2_, %	58 (53, 62)	61 (54, 68)	0.192

*Values are expressed as medians (interquartile range), except for female gender and WHO functional class, which are numbers of patients (%)*.

a *Comparison between groups with Ec-SOD ≤ 83 U/L and Ec-SOD > 83 U/L*.

b *6MWD could be successfully measured in 124 patients*.

*BMI, body mass index; BMPR 2, bone morphogenetic protein receptor type 2; BNP, brain natriuretic peptide; CI, cardiac index; CO, cardiac output; Cu/Zn-SOD, copper–zinc superoxide dismutase; Ec-SOD, extracellular form of Cu/Zn superoxide dismutase; HR, heart rate; Mn-SOD, manganese superoxide dismutase; mPAP, mean pulmonary arterial pressure; mRAP, mean right atrial pressure; 6MWD, 6-min walking distance; PAWP, pulmonary artery wedge pressure; PVR, pulmonary vascular resistance; SvO_2_, mixed venous oxygen saturation; WHO FC, World Health Organization functional class*.

## Discussion

Antioxidant enzymes play prominent roles against oxidative damage and provide protective signals in pathological remodeling of the pulmonary vasculature during the development of PAH. Understanding the SOD pathway will greatly help in the clinical development of medications for PAH therapy. However, it is unclear whether an abnormality of SOD activity is associated with hemodynamic dysfunction and poor outcomes in patients with IPAH. Our results demonstrate that (a) the plasma SOD activity was markedly reduced in patients with IPAH compared to that in healthy subjects, (b) patients with *BMPR2* mutation had a lower Ec-SOD level than the no-mutation subjects, (c) only Ec-SOD activity was correlated with hemodynamic abnormalities and survival, and (d) a decreased Ec-SOD activity was associated with an increased mortality risk, suggesting that the use of Ec-SOD may be superior to Cu/Zn-SOD or Mn-SOD for supplemental treatment if possible.

Oxidative stress in PAH is rather inescapable with the increased lipid peroxidation and reduced antioxidant defenses (20). The expression and the activity of SODs presumably have profound effects on the responses of vascular cells to oxidative stress (4). It is noteworthy that the plasma activity of all SODs' isoforms is statistically decreased in patients with IPAH compared with that in the healthy subjects in our study, especially for Ec-SOD activity. The decreases in Ec-SOD activities might not be attributed solely to superoxide or ROS level; the mechanisms of post-translation modification, genetic polymorphisms, and epigenetic regulation are included (21–23). For example, exogenous hydrogen peroxide (H_2_O_2_) inactivates Ec-SOD in persistent pulmonary hypertension of newborn lambs. Through oxidation of histidine residues in copper-containing catalytic sites, H_2_O_2_ has been shown to inhibit Ec-SOD activity (21). Moreover, Nozik-Grayck et al. have reported that histone deacetylation contributed to low Ec-SOD expression in PASMC from IPAH patients, specifically *via* class I histone deacetylase 3 (9, 23). There is also evidence that miR21 could inhibit Ec-SOD expression in lung epithelial cells in PAH (23). Collectively, Ec-SOD activity may be regulated by comprehensive processes in pulmonary hypertension; substantial work is geared on how to use Ec-SOD to evaluate the oxidative stress system.

Despite important advances in understanding the genetics of PAH (such as mutation in *BMPR2* in familial PAH) and the recognition of somatic chromosomal abnormalities in sporadic PAH, the cause of most cases of PAH remains yet unclear (24, 25). It has been reported that patients with *BMPR2* mutations exhibited a reduced level of NO production (26). Despite finding 21% of IPAH patients to have *BMPR2* mutations, not all patients with a mutation in the present cohort had a corresponding lower activity of SODs. It has been shown that Ec-SOD is the only extracellular isoform and occurs in bodily fluids such as plasma, lymph, and synovial and cerebrospinal fluid in the human organism (27). Ec-SOD can bind to the surface of endothelial cells by a high abundance of heparin sulfate. Consequently, it is highly expressed in lung tissue (28). Notably, Ec-SOD is the predominant isoform responsible for up to 70% of all SOD activity in the cardiovascular system (29, 30). Xu et al. reported that Ec-SOD gene mutation (SOD 3^*E*124*D*^) in rats or SOD 3 knockout in mice aggravated the development of PAH under stress conditions (10). In addition, Ec-SOD preserves NO levels as it diffuses from the endothelium to its major target (soluble guanylate cyclase) in the vascular muscle (4, 31). The activity of Ec-SOD was markedly lower in *BMPR2* mutation patients in our present study, possibly due to the decreased bioavailability of NO and/or decreased responsiveness to NO in PAH.

We found that the reduced Ec-SOD activity was closely associated with the severity of hemodynamic impairment in the study population as a whole, implying that antioxidant enzyme deficiency might partly reflect pulmonary vascular resistance under oxidative stress status. ROS could stimulate vasoconstriction or proliferation of PASMC through NO reduction, leading to the pathogenesis of PAH (12). Ec-SOD might halt the ROS cascade by disproportioning superoxide anion and maintaining NO bioavailability in pulmonary arteries (12). Our group has reported that patients with IPAH who carry the *BMPR2* mutation had further reduced NO metabolites and worse hemodynamics (27). Sustained Ec-SOD expression in the pulmonary artery might exert a central role in extracellular antioxidative properties (32). Hence, the low NO availability perhaps disturbs the balance and the distribution of SODs.

The role of SODs in pulmonary vasculature has not been fully understood. In a competing reaction, superoxide reacts six times faster with NO than with any isoform of the SODs (33, 34). The endogenous Ec-SOD does not participate in the development of PAH under basal conditions but plays a role of protecting the lung from the development of PAH under stress conditions (9). Reports of the association of genes encoding the SOD enzymes in cardiovascular complications are scarce (35). One study found a rare functional variant rs1799895 (Arg213Gly) in the heparin-binding domain of Ec-SOD. The Gly allele was associated with reduced Ec-SOD affinity for heparin and decreased tissue binding (36). Accordingly, the associations of the Gly allele with cardiovascular risk factors, morbidity, or mortality have been reported (35, 37, 38). Although we did not design and detect the genetic variant of Ec-SOD in patients with IPAH, it is an important finding that Ec-SOD, but not Cu/Zn-SOD or Mn-SOD levels, could predict long-term outcomes in our study as the strength of risk prediction for Ec-SOD activity was robust. The vascular wall contains large amounts of Ec-SOD, implicating that reduced Ec-SOD activity might contribute to endothelial dysfunction and NO degradation. Thus, SODs, which are responsible for preventing oxidative damage, might be beneficial for the supplemental treatment for IPAH.

Several limitations of the present study must be noted while interpreting the results. First, since plasma SOD levels were only measured at baseline, it was difficult for us to evaluate the variability and the prognosis of level changes over time or the impact of different therapies. Second, blood samples for measuring the plasma SOD levels were collected after an overnight (>12 h) fast in all patients and subjects; the best time to collect samples for prediction remains unclear. However, there are studies reporting that SOD activities were stable in samples initially kept frozen, whose activities were not subject to protein alteration as a result of the freezing procedure (39). The difference between inter-batch and inter-operator variability was reasonable. Furthermore, another potential limitation is lack of race; the findings may be applicable to the Chinese Han population. It remains to be explained whether the results of this study are exclusive to the selected population. Finally, we had better to perform the sensitivity analyses in order to assess the validity and the robustness of the analyses based on the cutoff value.

## Conclusion

In summary, we demonstrated that the baseline plasma SOD activities were significantly lower in patients with IPAH than in healthy control subjects. Only the Ec-SOD level was associated with the hemodynamic measures of disease severity and *BMPR2* mutation. Patients with reduced Ec-SOD activity had increased risks for mortality independent of clinical characteristics and other risk factors. Decreased circulating Ec-SOD could potentially be used as a biomarker in the prognosis of IPAH patients.

## What Is Already Known on This Subject?

▶ Antioxidant enzymes play prominent roles against oxidative damage and protective signals in pathological remodeling of the pulmonary vasculature during the development of idiopathic pulmonary arterial hypertension (IPAH). Superoxide dismutases (SODs) are an important family of antioxidant enzymes that modulate reactive oxygen species levels.

## WHat Might This Study Add?

▶ This study adds to the present knowledge that plasma SOD activities were significantly lower in patients with IPAH than in healthy control subjects. Only Ec-SOD level was associated with the hemodynamic measures of disease severity and BMPR2 mutation. Patients with reduced Ec-SOD activity had increased risks for mortality, independent of clinical characteristics and other risk factors. Ec-SOD is a vital antioxidant enzyme and superior to the other two isoforms in PAH pathogenesis.

## How Might This Impact on Clinical Practice?

▶ Understanding the SOD pathway will greatly help in the clinical development of medications for PAH therapy. This study demonstrated that the use of Ec-SOD may be superior to the other two SOD isoforms for supplemental treatment.

## Data Availability Statement

All datasets presented in this study are included in the article/Supplementary Material.

## Ethics Statement

The studies involving human participants were reviewed and approved by Shanghai Pulmonary Hospital Ethics Committee. The patients/participants provided their written informed consent to participate in this study.

## Author Contributions

All authors participated in the design of this study and/or patient enrolment and met the criteria for authorship. Z-CJ contributed to the study design, conduct of the study, data analysis, scientific overview, and editing of the manuscript and was directly involved in the recruitment and care of the participants. RZ contributed to the data analysis, scientific interpretation, and drafting and editing of the manuscript. LW, Q-HZ, RJ, S-GG, XJ, X-QX, Y-YH, and YL were directly involved in the recruitment and care of the participants and in data collection. All the authors had full access to all data of this study and had final responsibility for the decision to submit for publication. All the authors have reviewed the manuscript and approved the final version for submission.

## Conflict of Interest

The authors declare that the research was conducted in the absence of any commercial or financial relationships that could be construed as a potential conflict of interest.

## Abbreviations

BMI: body mass index
*BMPR2*: bone morphogenetic protein receptor type 2
BNP: brain natriuretic peptide
CI: cardiac index
95% CI: 95% confidence interval
CO: cardiac output
Cu/Zn-SOD: copper–zinc superoxide dismutase
Ec-SOD: extracellular form of Cu/Zn superoxide dismutase
IPAH: idiopathic pulmonary arterial hypertension
Mn-SOD: manganese superoxide dismutase
mPAP: mean pulmonary arterial pressure
mRAP: mean right atrial pressure
6MWD: 6-min walk distance
PAWP: pulmonary artery wedge pressure
PVR: pulmonary vascular resistance
ROC: receiver operation characteristic
SOD: superoxide dismutase
SvO_2_: mixed venous oxygen saturation.
