# Supplementary material for: Alteration of Extracellular Superoxide Dismutase in Idiopathic Pulmonary Arterial Hypertension
Source: Front Med (Lausanne). 2020 Nov 17;7:509. doi: 10.3389/fmed.2020.00509 (PMC7705200; doi:10.3389/fmed.2020.00509)

SUPPLEMENTARY FIGURES AND LEGENDS

SUPPLEMENTARY FIGURE 1. Correlation between age and plasma SODs values.

(A) Cu/Zn-SOD (r = -0.05, p = 0.58), (B) Mn-SOD (r = -0.07, p = 0.47), (C) Ec-SOD (r = -0.25, p = 0.07)

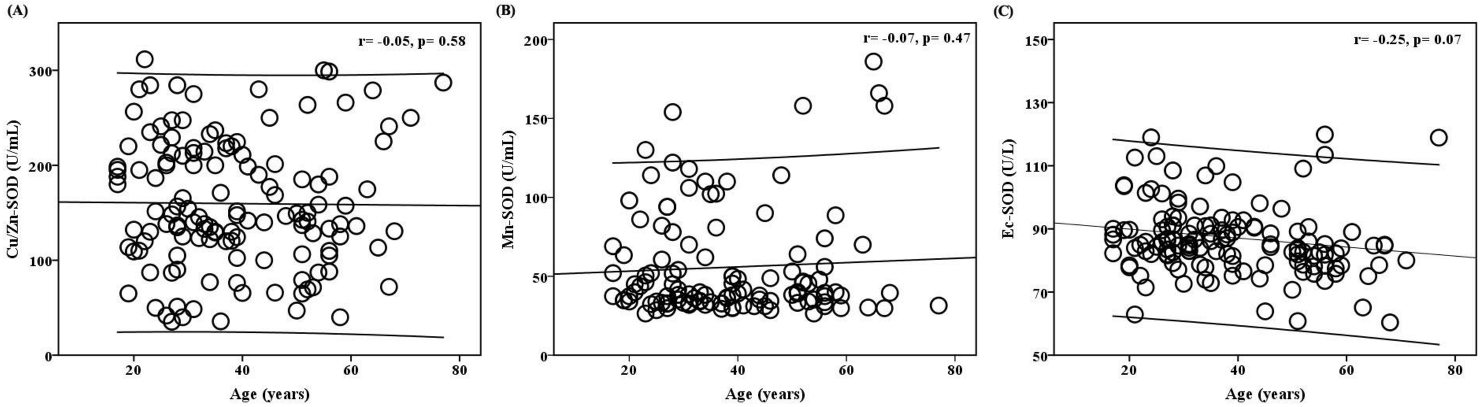

SUPPLEMENTARY FIGURE 2. Correlation between 6MWD and plasma Cu/Zn-SOD value (r = 0.03, p = 0.78).

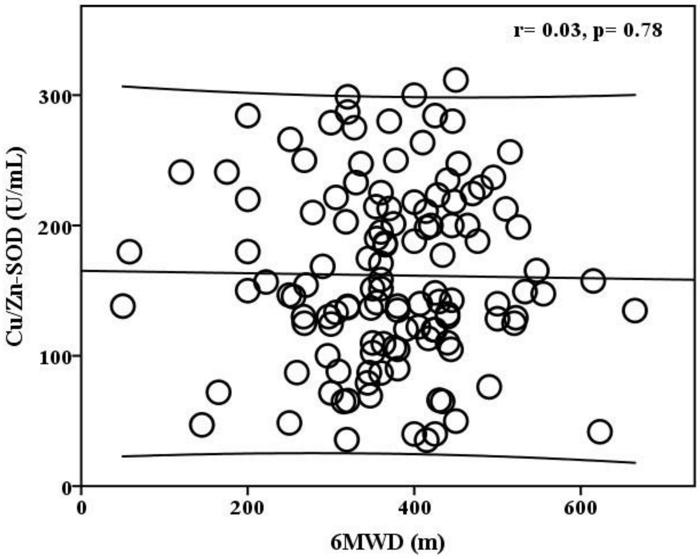

SUPPLEMENTARY FIGURE 3. Correlation between mPAP and plasma SODs values. (A) Cu/Zn-SOD (r = -0.02, p = 0.86), (B) Mn-SOD (r= -0.07, p= 0.49).

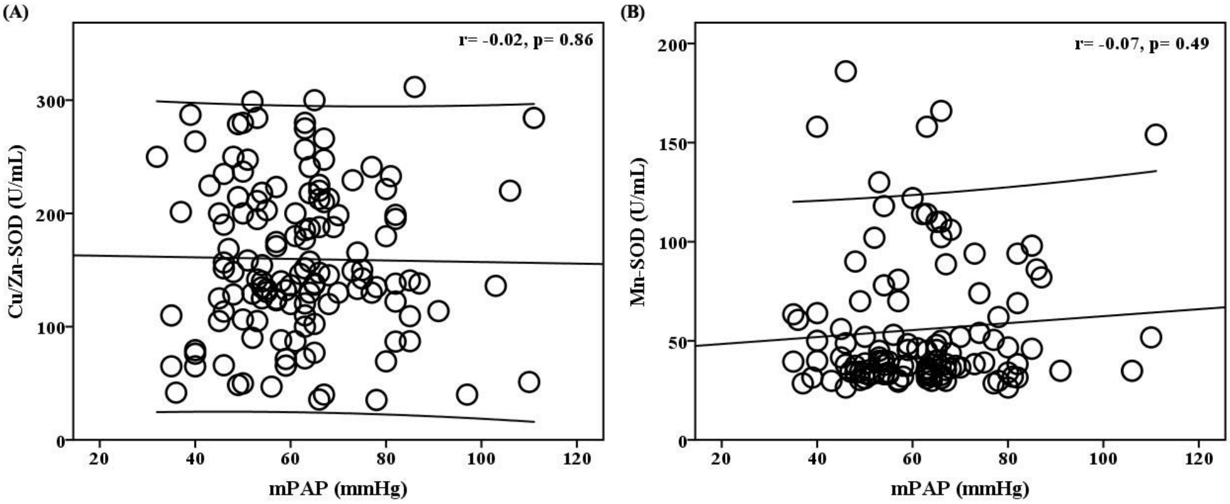

SUPPLEMENTARY FIGURE 4. Correlation between PVR and plasma SODs values.

(A) Cu/Zn-SOD (r = -0.07, p = 0.42), (B) Mn-SOD (r= -0.16, p= 0.10).

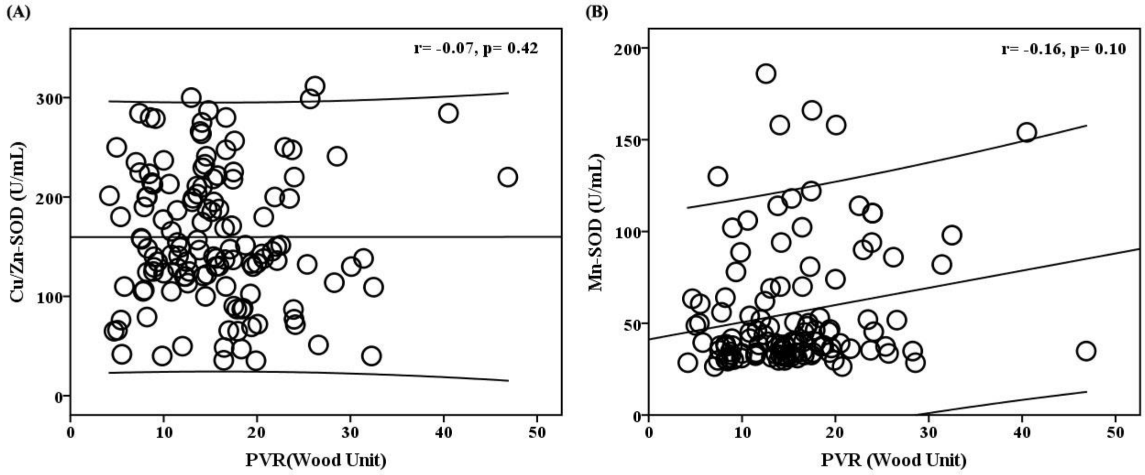

SUPPLEMENTARY FIGURE 5. Correlation between mRAP and plasma SODs values. (A) Cu/Zn-SOD (r = -0.02, p = 0.78), (B) Mn-SOD (r = -0.03, p = 0.72), (C) Ec-SOD (r = -0.06, p = 0.48)

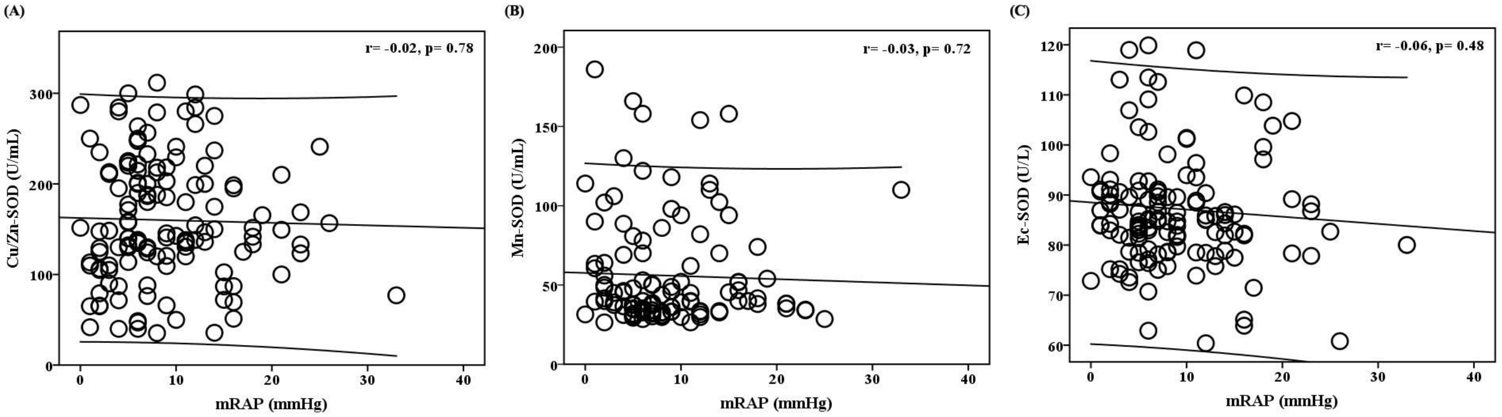

SUPPLEMENTARY FIGURE 6. Correlation between CO and plasma SODs values. (A) Cu/Zn-SOD (r = 0.04, p = 0.67), (B) Mn-SOD (r = 0.18, p = 0.16), (C) Ec-SOD (r = 0.07, p = 0.29)

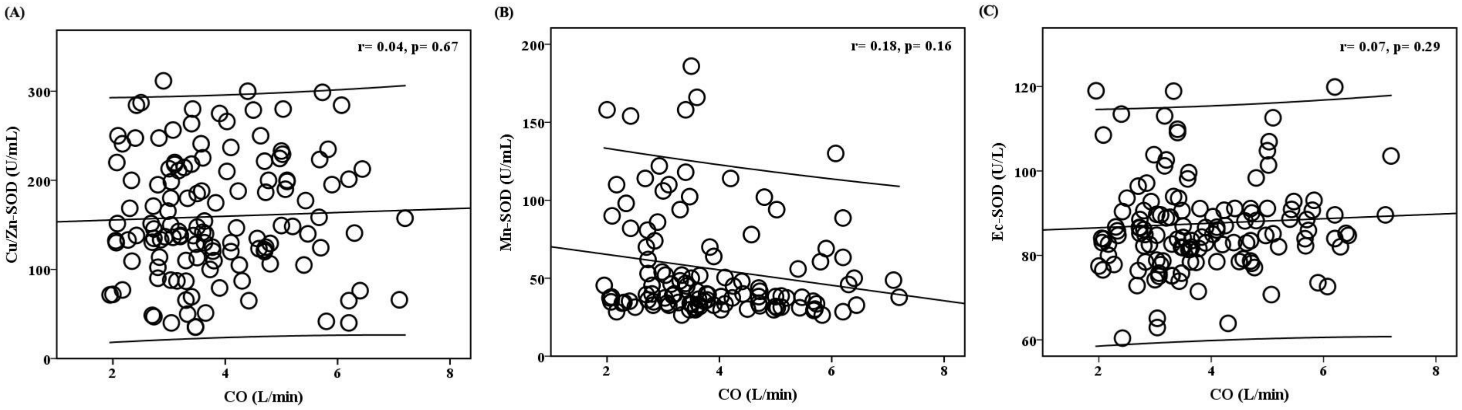

SUPPLEMENTARY FIGURE 7. Correlation between CI and plasma SODs values. (A) Cu/Zn-SOD (r = 0.05, p = 0.55), (B) Mn-SOD (r = 0.14, p = 0.12), (C) Ec-SOD (r = 0.03, p = 0.78)

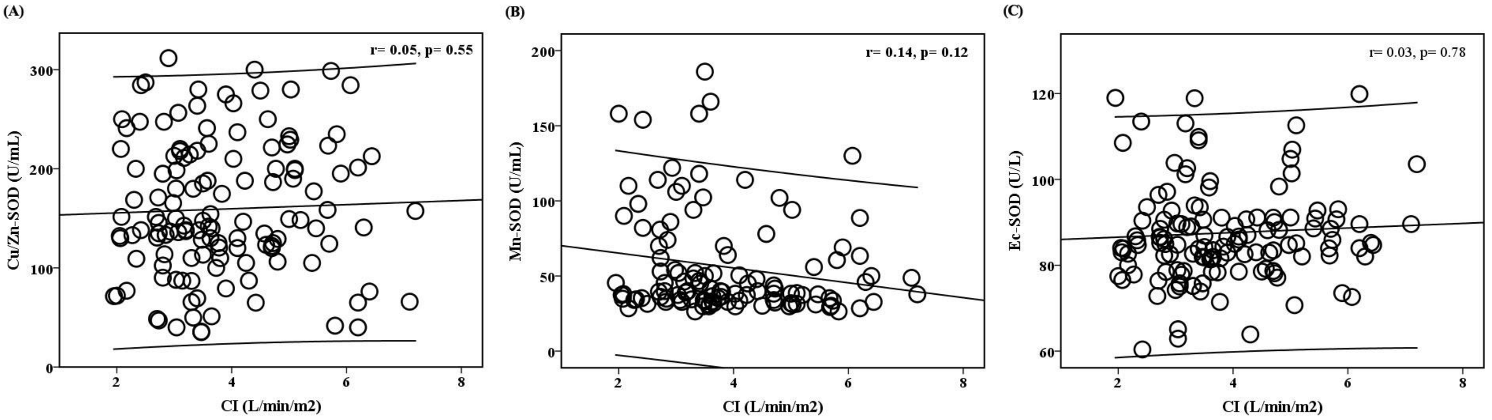

SUPPLEMENTARY FIGURE 8. Correlation between $SvO_2$and plasma SODs values. (A) Cu/Zn-SOD (r = 0.05, p = 0.58), (B) Mn-SOD (r = -0.02, p = 0.83), (C) Ec-SOD (r = 0.12, p = 0.17)

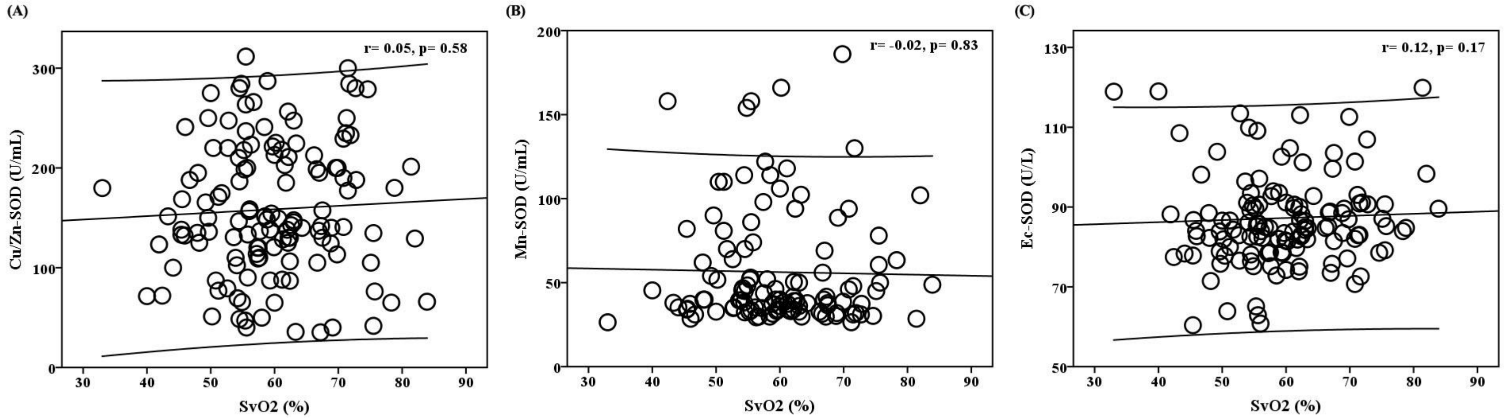

Supplement: Supplementary file 1 [file Data_Sheet_1.PDF]
